# Gastric infusion of short-chain fatty acids can improve intestinal barrier function in weaned piglets

**DOI:** 10.1186/s12263-019-0626-x

**Published:** 2019-02-01

**Authors:** H. Diao, A. R. Jiao, B. Yu, X. B. Mao, D. W. Chen

**Affiliations:** 1 Institute of Animal Nutrition, Sichuan Agricultural University, Xinkang Road 46#, Ya’an, 625014 Sichuan Province People’s Republic of China; 2 Animal Breeding and Genetics key Laboratory of Sichuan Province, Sichuan Academy of Animal Science, Chengdu, Sichuan Province People’s Republic of China

**Keywords:** Short-chain fatty acids, Intestinal growth, Apoptotic, Gut barrier, Piglets

## Abstract

**Background:**

The present study was conducted to investigate the effects of gastric infusion of short-chain fatty acids (SCFA) on gut barrier function in a pig model. In this study, 21 DLY barrows with an average initial body weight of (8.31 ± 0.72) kg were randomly allotted into three treatments: (1) control, (2) infusing low SCFA, S1, (3) infusing high SCFA, S2. The experimental period lasted for 7 days.

**Results:**

Gastric infusion of SCFA increased the concentrations of SCFA in serum and digesta, and enhanced the mRNA and protein abundances of SCFA receptors in pig intestine (*P* < 0.05). Moreover, gastric infusion of SCFA led to alteration of intestinal morphology, elevation of intestinal development-related gene abundances, and decrease of apoptotic cell percentage, as well as reduction of pro-apoptosis gene and protein abundances (*P* < 0.05). Besides, the jejunal SLC_7_A_1_ and ileal DMT1 mRNA abundances in the SCFA infusion groups were higher than those in the control group (*P* < 0.05). Additionally, gastric infusion of SCFA increased the mRNA abundances of Occludin and Claudin-1 in the duodenum and ileum, enhanced *Lactobacillus* spp counts in the ileal digesta, decreased the mRNA and protein abundances of IL-1β in the colon, and reduced *Escherichia coli* count in the ileal digesta (*P* < 0.05).

**Conclusions:**

These data indicated that gastric infusion of SCFA, especially high SCFA concentration, may be beneficial to gut development of piglets via improving gut morphology, decreasing apoptotic cell percentage, and maintaining intestinal barrier function.

**Electronic supplementary material:**

The online version of this article (10.1186/s12263-019-0626-x) contains supplementary material, which is available to authorized users.

## Introduction

Hindgut fermentation is a phenomenon which is ubiquitous to all mammals [1]. Short-chain fatty acids (SCFA, such as acetate, propionate, and butyrate), the metabolites of gut microbiota fermentation, have vital physiological functions. Acetate and propionate are largely metabolized in the liver and are involved in lipid and glucose metabolism, respectively, while butyrate is shown to supply energy for the epithelial cells of colon [2, 3]. Usually, the proportions of acetate, propionate, and butyrate in the intestinal content are 60:25:15, and the SCFA profile can mirror the metabolic cooperation among microorganisms [4].

Intestinal epithelium is usually recognized as one of the most rapid proliferation tissues in animals, and its turnover rate is 2 to 3 days [5, 6]. Previous studies have shown that SCFA are involved in intestinal epithelial cell proliferation, but the effects of SCFA on intestinal epithelial cell proliferation are controversial in in vivo and in vitro studies. Studies in rats revealed that ileal or colonic infusion of butyrate or a combination of SCFA mixture increased intestinal crypt cell production rate and enhanced the mucosal weight and the concentrations of mucosal DNA, RNA, and protein [7, 8]. Similarly, the sole study in a pig model demonstrated cecal infusion of butyrate stimulated epithelial cell proliferation index and promoted intestinal cell proliferation [9]. However, the in vitro studies found inconsistent results, which revealed the inhibitory effect on epithelial proliferation and cell viability under the treatment of SCFA [7, 10]. Therefore, the controversial effect of SCFA on cell proliferation needs to be further investigated.

Intestinal mucosa is consisted of epithelial cells and responsible for nutrient absorption and waste secretion, which requires a selectively permeable barrier. Hence, the integrity of gut barrier function is required for intestinal health [11]. Recently, many in vitro studies have shown that SCFA may mediate the intestinal barrier function. Propionic acid inhibited *Staphylococcus aureus* internalization into bovine mammary epithelial cells in vitro [12], while butyrate promoted intestinal barrier function as measured by increasing the relative mRNA expression of tight junction and their re-assembly as well as elevating transepithelial electrical resistance (TER) in Caco2 and IPEC-J2 cells [13, 14]. Besides, the increasing relative mRNA expressions of MUC-2, MUC-3, MUC-4, and MUC-12 were observed in LS174T human colorectal cells with the presence of butyrate [15]. Meanwhile, SCFA could downregulate the pro-inflammatory cytokines expressions in Caco-2 cells under LPS challenge [16]. Additionally, the in vivo studies also revealed that dietary sodium butyrate supplementation could maintain intestinal barrier via reducing the IL-6 and TNF-a levels in the serum, decreasing the number of *Clostridium* and *Escherichia coli*, as well as increasing the number of *Lactobacillus* spp in pigs [17, 18].

The integrity of intestinal epithelium is closely related to gut health, and the intestinal redox status (antioxidant capacity) can affect intestinal epithelial integrity [11]. In the normal physiological condition, the digestive tract can generate reactive oxygen species (ROS) [19]. However, numerous factors, such as weaning, infection, and environmental impacts, can induce oxidative stress, resulting in imbalance between the reactive oxygen species (ROS) concentrations and intra- or extracellular antioxidants, which brings serious economic losses during livestock production [20, 21]. In response to the injury of free radicals, there are enzymatic and non-enzymatic antioxidant systems existing in body, and the enzymatic antioxidant system mainly consists of GSH-px and SOD [22]. A recent in vitro study demonstrated that butyrate could upregulate the GPx-3, GPx-4, and total GPx mRNA expressions in vascular smooth muscle cell [23]. So, the in vivo study of SCFA on intestine antioxidant capacity still needs to be further investigated.

Remarkably, it is easy to cause intestinal stress when piglets are transferred to feed from sucking milk after weaning, which is associated with morphological and physiological alterations, including intestinal villous atrophy, crypt hyperplasia, and destroyed epithelial barrier [24]. However, the systematic crosstalk of SCFA and intestinal barrier function in vivo model has been rarely investigated, especially in pig models, and whether SCFA can attenuate the weaning stress action or not is unknown. Taking these into consideration, the objective of present study was to systematically evaluate the effects of gastric infusion of different concentrations of SCFA on intestinal structure and functions in weaned piglets, which could help us to further understand the underlying mechanisms of the regulation role of SCFAs on intestinal development.

## Results

### Short-chain fatty acids and their receptors

**Fig. 1:**
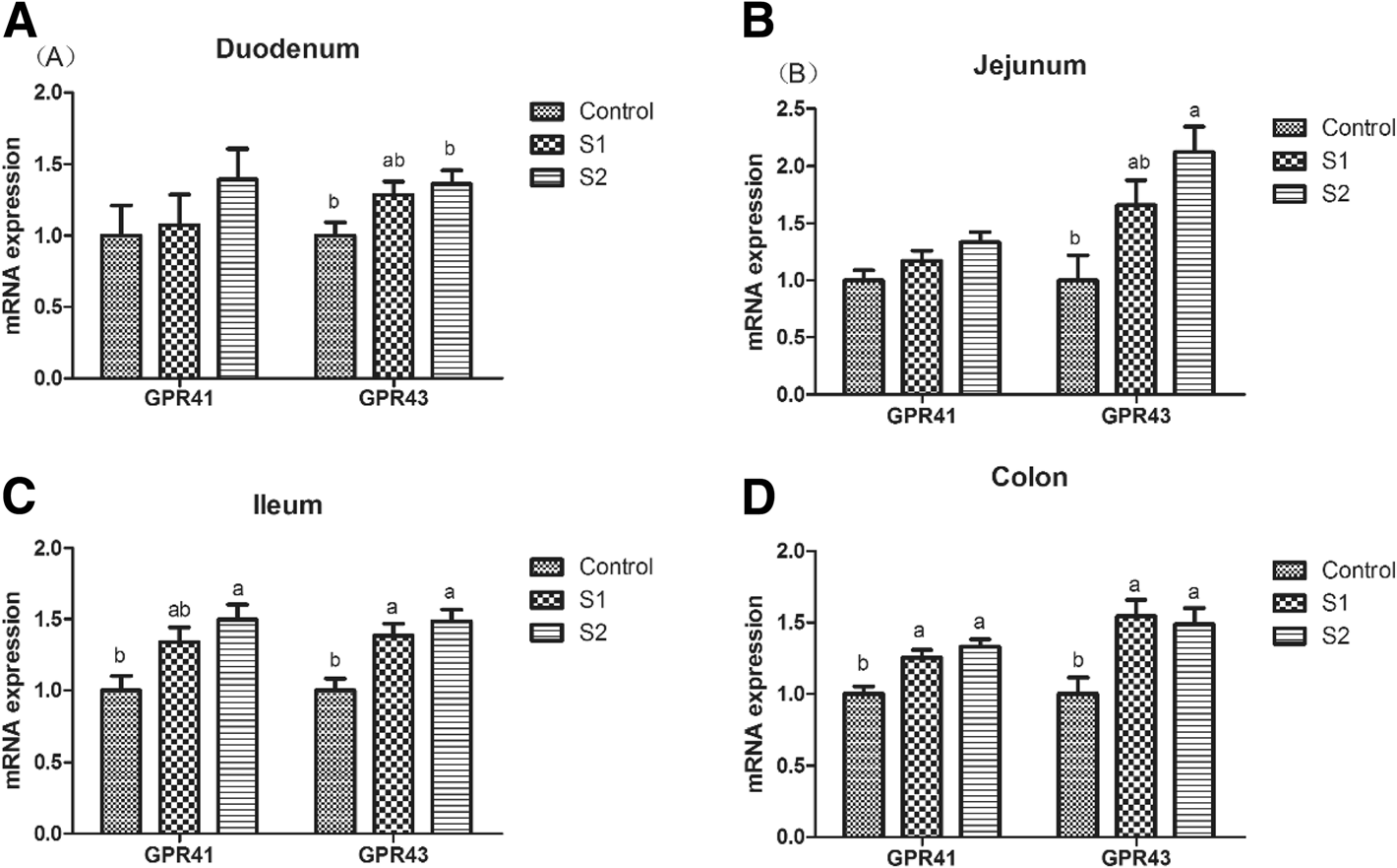
Effect of gastric infusion of SCFA on the relative mRNA expressions of SCFA receptors in ileum and colon of growing pigs. S1, pigs treated with SCFA (acetic, propionic, and butyric acids; 20.04, 7.71, and 4.89 mM respectively); S2, pigs treated with SCFA (acetic, propionic, and butyric acids; 40.08, 15.41, and 9.78 mM respectively). a, b, means without a common superscript difference (*P* < 0.05)

As shown in Table 1, gastric infusion of SCFA increased the concentration of butyric acid in the serum, and the concentrations of acetic acid, propionic acid, butyric acid, and total SCFA in the ileal, cecal, and colonic digesta (*P* < 0.05). The contents of acetic acid, propionic acid, butyric acid, and total SCFA in the serum; the contents of acetic acid, propionic acid, and total SCFA in the ileal digesta; and the contents of butyric acid and total SCFA in the colonic digesta in the S2 group were higher than those in the S1 group (*P* < 0.05). As shown in Fig. 1, gastric infusion of SCFA was associated with relatively higher mRNA expression of GPR41 in the colon and GPR43 in the ileum and colon compared with the control group (*P* < 0.05). Meanwhile, the GPR43 protein level of jejunum and colon in the S1 and S2 groups were higher than that in the control group, and pigs of S2 group had higher GPR43 protein level in the jejunum than pigs of S1 group (Figs. 7 and 8, *P* < 0.05).

**Table 1:** Effect of gastric infusion of SCFA on SCFA concentration in ileal, cecal, colonic digesta, and serum of weaned piglets (μmol/g)

Items	Control	S1	S2	SEM	*P* value
Serum (mmol/l)					
Acetic acid	0.733^b^	0.947^b^	1.553^a^	3.363	0.001
Propionic acid	0.622^b^	0.871^b^	1.292^a^	0.088	0.001
Butyric acid	0.236^c^	0.345^b^	0.420^a^	0.075	< 0.001
Total volatile fatty acid	1.591^b^	2.162^b^	3.265^a^	0.013	0.001
Ileum					
Acetic acid	3.667^c^	9.083^b^	12.011^a^	0.116	< 0.001
Propionic acid	1.119^c^	1.788^b^	1.975^a^	0.381	< 0.001
Butyric acid	0.377^b^	0.697^a^	0.747^a^	0.037	< 0.001
Total volatile fatty acid	5.163^c^	11.568^b^	14.733^a^	0.021	< 0.001
Cecum					
Acetic acid	37.735^b^	55.438^a^	58.801^a^	0.383	< 0.001
Propionic acid	17.893^b^	28.223^a^	31.868^a^	1.774	0.001
Butyric acid	11.296^b^	17.073^a^	20.108^a^	1.449	0.002
Total volatile fatty acid	66.923^b^	100.734^a^	110.778^a^	1.249	< 0.001
Colon					
Acetic acid	33.898^b^	44.368^a^	51.732^a^	3.297	0.001
Propionic acid	14.343^b^	24.481^a^	27.022^a^	1.972	< 0.001
Butyric acid	8.115^c^	18.836^b^	23.675^a^	1.252	< 0.001
Total volatile fatty acid	56.356^c^	87.684^b^	102.429^a^	1.043	< 0.001

S1, pigs treated with SCFA (acetic, propionic, and butyric acids; 20.04, 7.71, and 4.89 mM respectively); S2, pigs treated with SCFA (acetic, propionic, and butyric acids; 40.08, 15.41, and 9.78 mM respectively)

^a, b, c^Within a row, means without a common superscript differ (*P* < 0.05)

### Intestinal index and pH value

Compared with the control group, pigs treated with gastric SCFA infusions had lower pH value of digesta in the duodenum and colon (Table 2, *P* < 0.05). The pH value of jejunal and ileal digesta in the S2 group was lower than that in the control group (*P* < 0.05). The relative length and weight of small intestine and total intestine in the S2 group were higher than those in the control group (*P* < 0.05).

**Table 2:** Effect of gastric infusion of SCFA on intestinal index and pH value of digesta in weaned piglets

Items	Control	S1	S2	SEM	*P* value
Intestinal index					
Relative length of SI (cm/g)	8.510^b^	8.706^ab^	9.132^a^	0.147	0.037
Relative length of LI (cm/g)	1.966	2.060	2.093	0.035	0.069
Relative length of I (cm/g)	10.476^b^	10.766^ab^	11.225^a^	0.164	0.027
Relative density of SI(g/cm)	0.460	0.503	0.517	0.023	0.226
Relative density of LI(g/cm)	0.800	0.824	0.829	0.022	0.622
Relative density of I(g/cm)	0.523	0.565	0.574	0.018	0.165
Relative weight of SI (%)	3.905^b^	4.381^ab^	4.710^a^	0.160	0.016
Relative weight of LI (%)	1.573	1.698	1.732	0.058	0.176
Relative weight of I (%)	5.478^b^	6.078^ab^	6.442^a^	0.170	0.008
pH values					
Stomach	3.825	3.593	3.293	0.324	0.531
Duodenum	5.308^a^	4.388^b^	4.157^b^	0.185	0.003
Jejunum	6.833^a^	6.773^ab^	6.293^b^	0.138	0.038
Ileum	6.954^a^	6.722^ab^	6.552^b^	0.093	0.037
Cecum	5.915	5.542	5.455	0.180	0.209
Colon	6.482^a^	5.845^b^	5.688^b^	0.111	0.001

S1, pigs treated with SCFA (acetic, propionic, and butyric acids; 20.04, 7.71, and 4.89 mM respectively); S2, pigs treated with SCFA (acetic, propionic, and butyric acids; 40.08, 15.41, and 9.78 mM respectively)

^a, b^Within a row, means without a common superscript differ (*P* < 0.05)

### Intestinal morphology

As shown in Table 3, gastric infusion of SCFA increased the villus height of jejunum and ileum in piglets (*P* < 0.05). The villus height of duodenum and crypt depth of duodenum, jejunum, and ileum in the S2 group were higher than those in the other two groups (*P* < 0.05). The villus height:crypt depth of duodenum and jejunum in the S1 group was higher than that in the control group (*P* < 0.05).

**Table 3:** Effect of gastric infusion of SCFA on intestinal morphology and number of goblet cell in weaned piglets

Items	Control	S1	S2	SEM	*P* value
Duodenum					
Villus height (μm)	332.47^b^	405.33^b^	522.47^a^	26.289	0.002
Crypt depth (μm)	149.51^b^	143.97^b^	228.12^a^	19.443	0.021
Villus height: crypt depth	2.23^b^	2.86^a^	2.40^ab^	0.142	0.028
Jejunum					
Villus height (μm)	244.96^b^	377.03^a^	415.04^a^	14.591	< 0.001
Crypt depth (μm)	152.68^b^	152.06^b^	222.28^a^	10.977	0.001
Villus height:crypt depth	1.62^b^	2.48^a^	1.91^b^	0.118	0.001
Ileum					
Villus height (μm)	208.48^b^	302.30^a^	346.17^a^	21.691	0.004
Crypt depth (μm)	157.45^b^	160.80^b^	227.87^a^	16.401	0.021
Villus height:crypt depth	1.35	1.90	1.54	0.148	0.070
Goblet cells					
Ileum	62.06^c^	88.33^a^	76.46^b^	2.825	< 0.001
Colon	51.81^b^	81.08^a^	89.39^a^	5.028	0.001

S1, pigs treated with SCFA (acetic, propionic, and butyric acids; 20.04, 7.71, and 4.89 mM respectively); S2, pigs treated with SCFA (acetic, propionic, and butyric acids; 40.08, 15.41, and 9.78 mM respectively)

^a, b, c^Within a row, means without a common superscript differ (*P* < 0.05)

### Intestinal cell apoptosis and cell cycle

The impacts of gastric SCFA infusion on intestinal cell apoptosis and cell cycle are shown in Table 4. Compared with the control group, S2 group had lower percentages of late apoptotic cells (*P* < 0.05) and total apoptotic cells (*P* = 0.063) in the jejunum, while S1 and S2 groups had lower percentages of late apoptotic cells and total apoptotic cells in the colon (*P* < 0.05). Moreover, gastric SCFA infusion decreased the ratio of G0G1 phase cells, but increased proliferation index in the jejunum and colon of pigs (*P* < 0.05). Besides, there was a tendency to increase the proportion of G2M phase cells of jejunum with SCFA infusion (*P* = 0.096). In addition, pigs of S2 group had lower ratio of G0G1 phase cells and higher proliferation index of jejunum than pigs of S1 group (*P* < 0.05).

**Table 4:** Effect of gastric infusion of SCFA on apoptosis and cell cycle in jejunum and colon of weaned piglets (%)

Items	Control	S1	S2	SEM	*P* value
Jejunum					
Early apoptotic cells	0.322	0.074	0.003	0.107	0.168
Late apoptotic cells	0.975^a^	0.268^ab^	0.007^b^	0.210	0.042
Total apoptotic cells	1.218	0.334	0.009	0.313	0.063
G0G1 phase cells	73.388^a^	63.092^b^	53.642^c^	1.969	< 0.001
S phase cells	18.108	21.897	29.512	3.569	0.119
G2M phase cells	8.502	15.015	16.848	2.536	0.096
PI	26.61^c^	36.911^b^	46.359^a^	1.969	< 0.001
Colon					
Early apoptotic cells	4.053	3.358	1.700	1.354	0.493
Late apoptotic cells	20.975^a^	7.220^b^	3.155^b^	2.032	0.002
Total apoptotic cells	26.219^a^	10.578^b^	4.855^b^	1.789	0.001
G0G1 phase cells	62.957^a^	55.125^b^	49.610^b^	1.855	0.002
S phase cells	30.163	33.785	37.463	2.261	0.123
G2M phase cells	6.883	11.093	12.922	2.411	0.241
PI	37.045^b^	44.877^a^	50.388^a^	1.855	0.002

S1, pigs treated with SCFA (acetic, propionic and butyric acids; 20.04, 7.71, and 4.89 mM respectively); S2, pigs treated with SCFA (acetic, propionic, and butyric acids; 40.08, 15.41, and 9.78 mM respectively)

PI = (S + G2M)/(G0G1 + S + G2M) × 100%

^a, b^Within a row, means without a common superscript differ (*P* < 0.05)

**Fig. 2:**
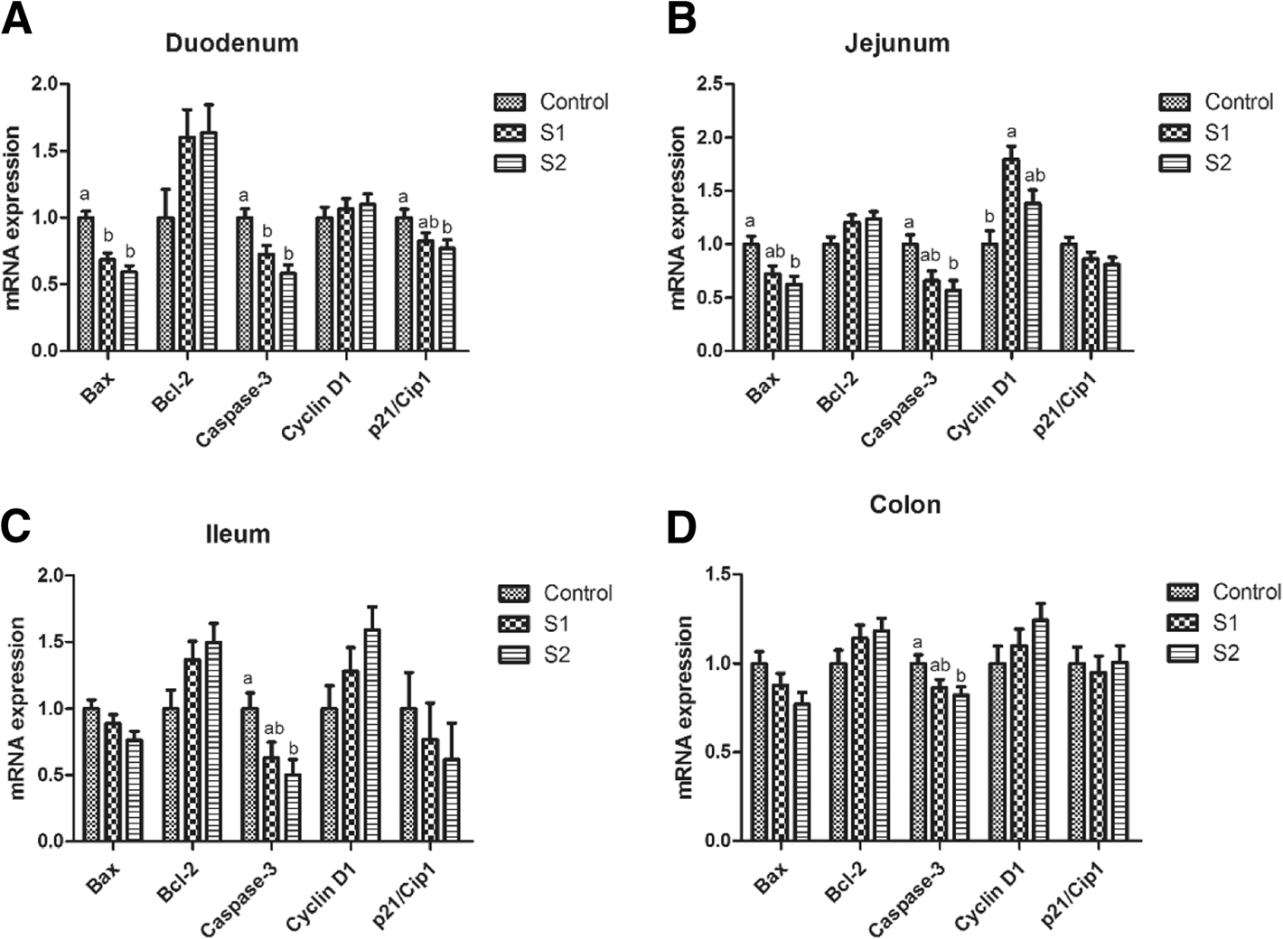
Effect of gastric infusion of SCFA on the relative mRNA expressions of cell apoptosis and cycle-related genes in duodenum, jejunum, ileum, and colon of weaned piglets. S1, pigs treated with SCFA (acetic, propionic, and butyric acids; 20.04, 7.71, and 4.89 mM respectively); S2, pigs treated with SCFA (acetic, propionic, and butyric acids; 40.08, 15.41, and 9.78 mM respectively). a, b, means without a common superscript differ (*P* < 0.05)

As shown in Fig. 2, gastric infusion of SCFA decreased the relative mRNA expressions of Bax and Caspase-3 in the duodenum compared with the control group (*P* < 0.05). S2 group exhibited lower relative mRNA expressions of Bax in the jejunum and Caspase-3 in the jejunum, ileum, and colon than control group (*P* < 0.05), while S1 group had higher relative mRNA expression of Cyclin D1 in the jejunum than control group (*P* < 0.05). In addition, the Caspase-3 protein levels of jejunum in S1 and S2 groups and colon in S2 group were lower than those in the control group, while pigs of S2 group had lower Caspase-3 protein level in the jejunum than pigs of S1 group (Figs. 7 and 8, *P* < 0.05).

### Intestinal DNA, protein, GLP-2 concentration, GLP-2R level, and relative mRNA expression of intestinal development-related gene

Compared with the control group, gastric SCFA infusion increased DNA concentration of jejunal mucosa and protein concentrations of duodenal and jejunal mucosa (Table 5, *P* < 0.05). Pigs of S2 group had higher protein concentration of ileal mucosa, GLP-2 concentration of jejunal mucosa, and DNA concentrations of duodenal, ileal, and colonic mucosa than pigs of control group (*P* < 0.05). Besides, pigs treated with gastric SCFA infusion had enhanced GLP-2R protein level in the jejunum and colon, and pigs of S2 group had higher GLP-2R protein level in the jejunum than pigs of S1 group (Figs. 7 and 8, *P* < 0.05).

**Table 5:** Effect of SCFA on DNA, protein, and GLP-2 concentration of mucosa in duodenum, jejunum, ileum, and colon of weaned piglets (μg/mg)

Items	Control	S1	S2	SEM	*P* value
DNA concentration					
Duodenum	0.946^b^	1.139^ab^	1.219^a^	0.052	0.012
Jejunum	0.984^b^	1.186^a^	1.299^a^	0.034	0.001
Ileum	1.027^b^	1.202^b^	1.453^a^	0.051	0.006
Colon	1.342^b^	1.447^ab^	1.576^a^	0.039	0.001
Protein concentration					
Duodenum	39.060^b^	44.622^a^	46.242^a^	0.891	0.001
Jejunum	39.924^b^	46.836^a^	47.664^a^	1.530	0.010
Ileum	39.906^b^	44.766^ab^	45.144^a^	1.269	0.027
Colon	34.740	38.565	39.942	2.493	0.350
GLP-2 concentration					
Jejunum (pmol/gprot)	1.036^b^	1.049 ^ab^	1.196^a^	0.048	0.073
Colon (pmol/gprot)	0.889	0.882	0.967	0.060	0.554

S1, pigs treated with SCFA (acetic, propionic, and butyric acids; 20.04, 7.71, and 4.89 mM respectively); S2, pigs treated with SCFA (acetic, propionic, and butyric acids; 40.08, 15.41, and 9.78 mM respectively)

^a, b^Within a row, means without a common superscript differ (*P* < 0.05)

**Fig. 3:**
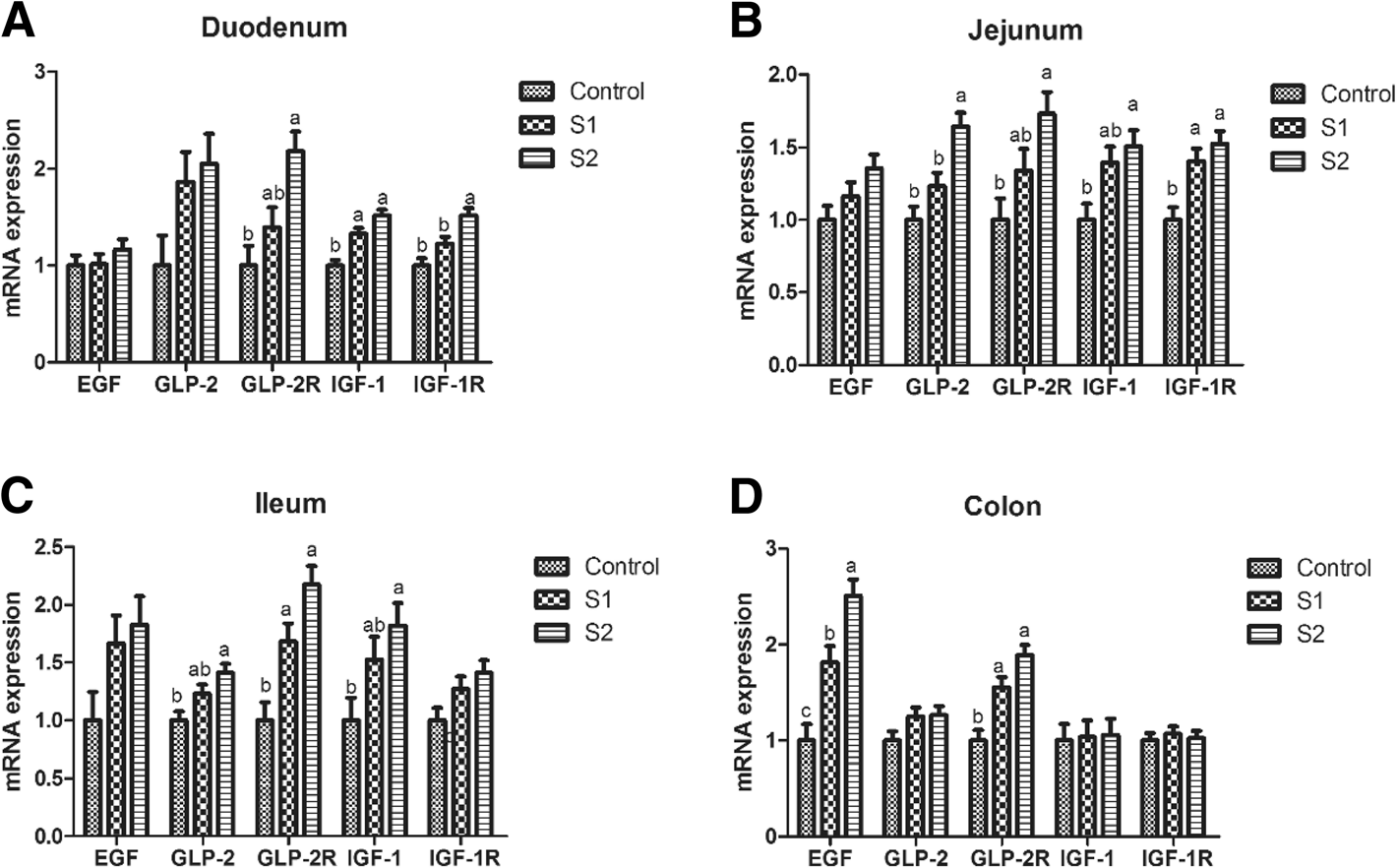
Effect of gastric infusion of SCFA on the relative mRNA expressions of intestinal development-related genes in duodenum, jejunum, ileum, and colon of weaned piglets. S1, pigs treated with SCFA (acetic, propionic, and butyric acids; 20.04, 7.71, and 4.89 mM respectively); S2, pigs treated with SCFA (acetic, propionic, and butyric acids; 40.08, 15.41, and 9.78 mM respectively). a, b, means without a common superscript differ (*P* < 0.05)

As shown in Fig. 3, pigs treated with gastric SCFA infusion had enhanced relative mRNA expressions of IGF-1 in the duodenum, IGF-1R in the jejunum, EGF in the colon, and GLP-2R in the ileum and colon relative to the control group (*P* < 0.05). S2 group had higher relative mRNA expressions of GLP-2 and IGF-1 in the jejunum and ileum, IGF-1R in the duodenum, and GLP-2R in the duodenum and jejunum than control group (*P* < 0.05). The relative mRNA expressions of IGF-1R in the duodenum, GLP-2 in the jejunum, and EFG in the colon in S2 group were higher than those in the S1 group (*P* < 0.05).

### Intestinal antioxidant capacity, goblet cell number, microbiota, and relative mRNA and protein expression of mucin, tight junction protein, and cytokine

**Fig. 4:**
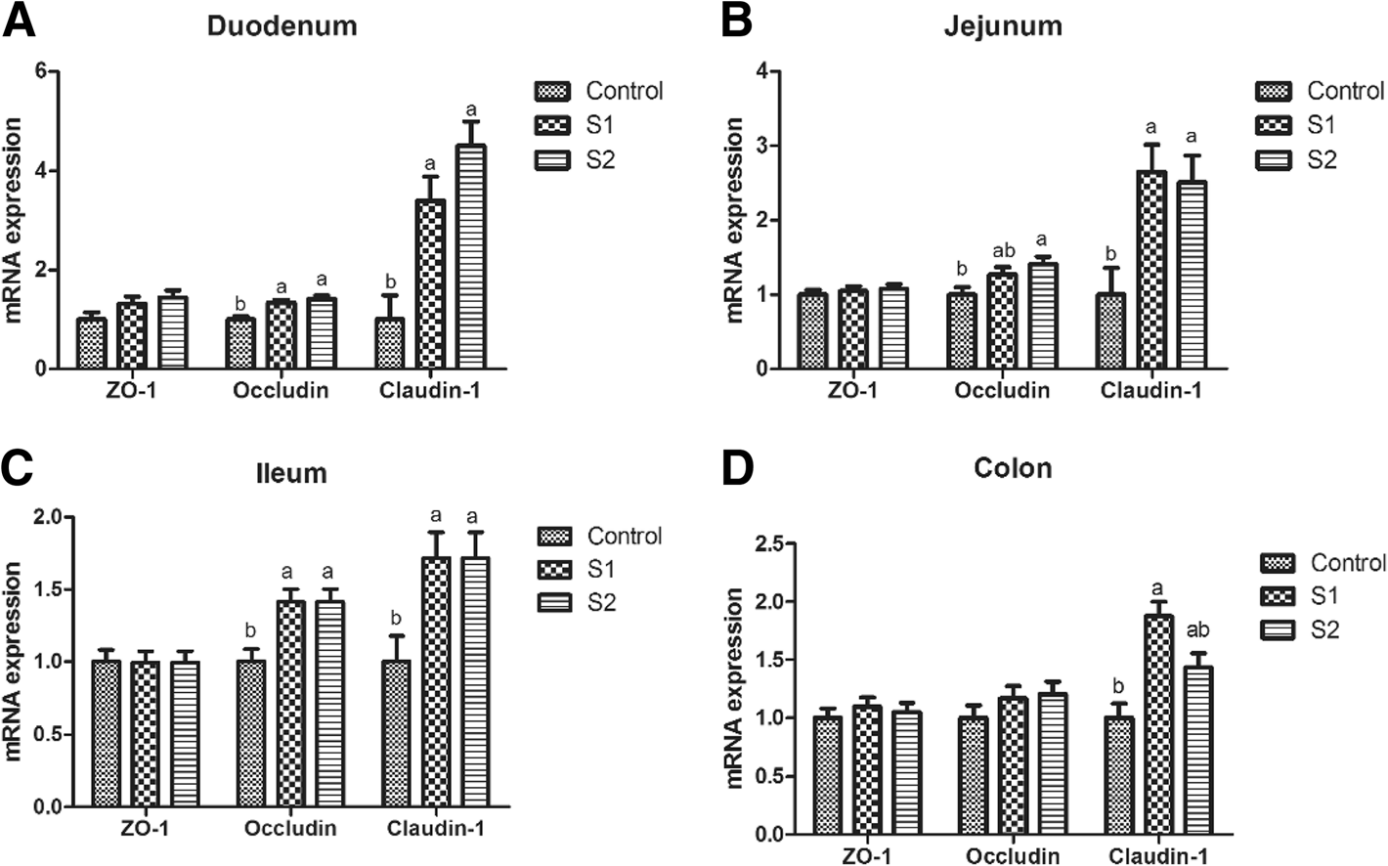
Effect of gastric infusion of SCFA on the relative mRNA expressions of intestinal tight junction-related genes in duodenum, jejunum, ileum, and colon of weaned piglets. S1, pigs treated with SCFA (acetic, propionic, and butyric acids; 20.04, 7.71, and 4.89 mM respectively); S2, pigs treated with SCFA (acetic, propionic, and butyric acids; 40.08, 15.41, and 9.78 mM respectively). a, b, means without a common superscript differ (*P* < 0.05)

**Fig. 5:**
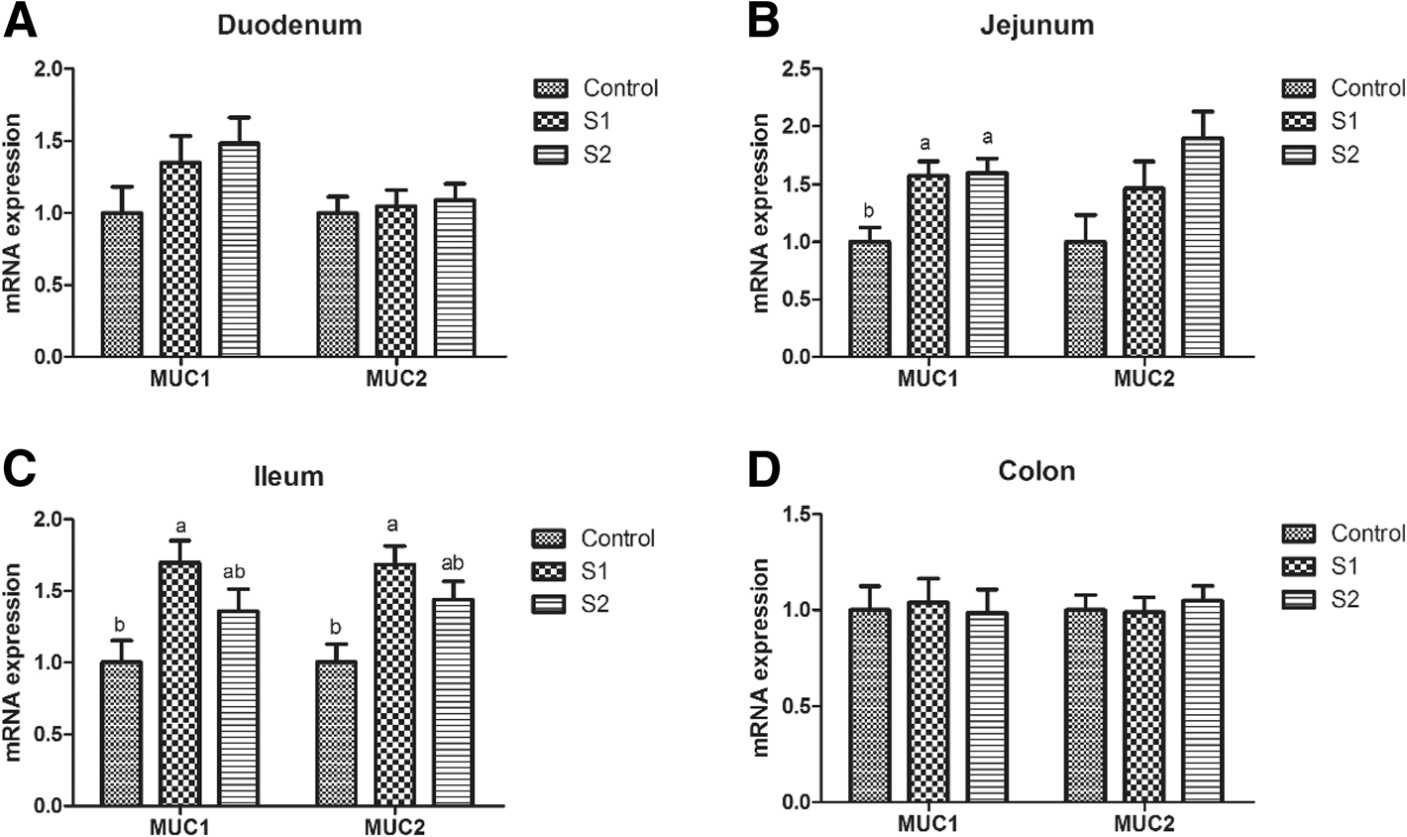
Effect of gastric infusion of SCFA on the relative mRNA expressions of mucin in duodenum, jejunum, ileum, and colon of weaned piglets. S1, pigs treated with SCFA (acetic, propionic, and butyric acids; 20.04, 7.71, and 4.89 mM respectively); S2, pigs treated with SCFA (acetic, propionic, and butyric acids; 40.08, 15.41, and 9.78 mM respectively). a, b, means without a common superscript differ (*P* < 0.05)

**Fig. 6:**
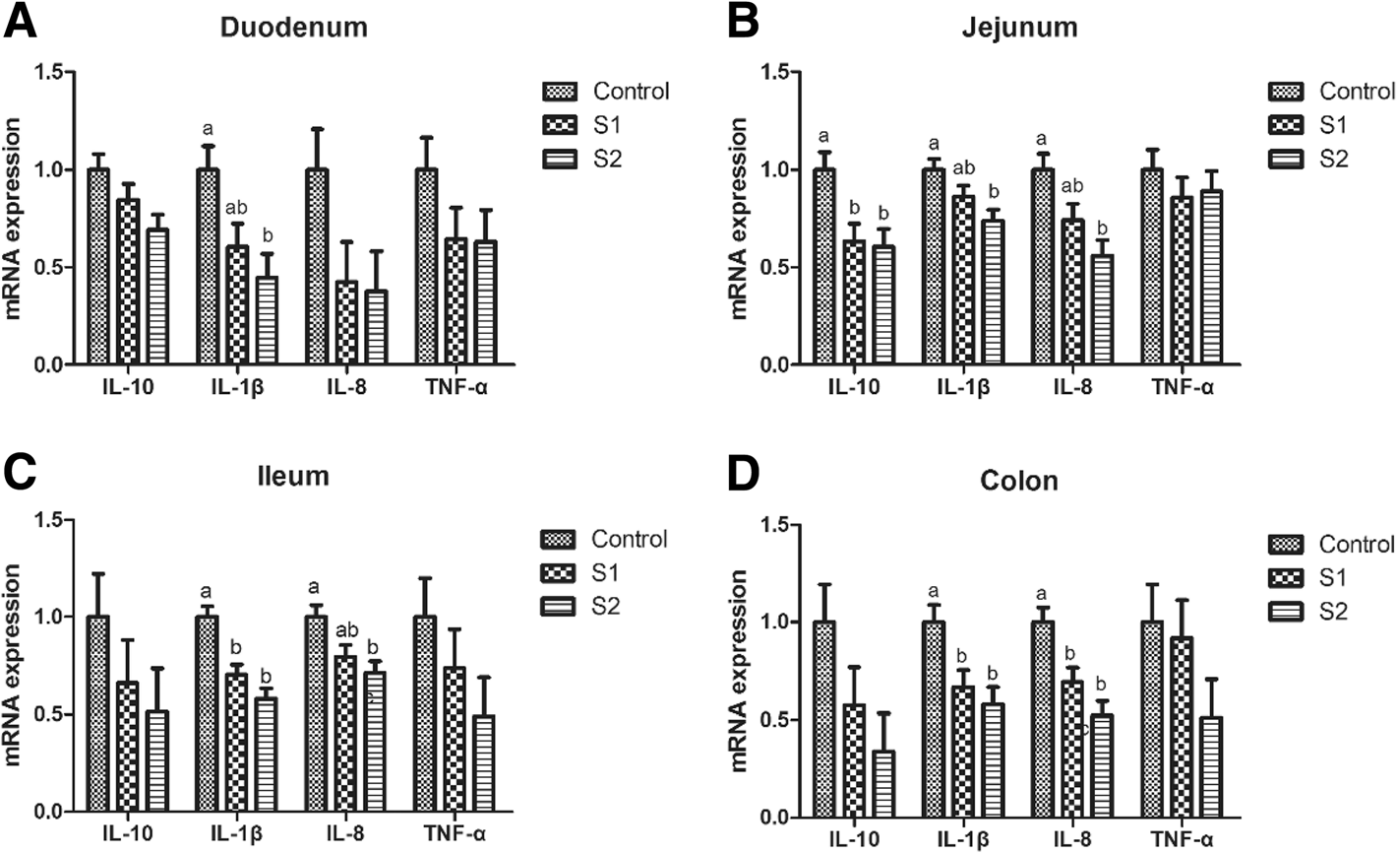
Effect of gastric infusion of SCFA on the relative mRNA expressions of inflammatory factors in duodenum, jejunum, ileum, and colon of weaned piglets. S1, pigs treated with SCFA (acetic, propionic, and butyric acids; 20.04, 7.71, and 4.89 mM respectively); S2, pigs treated with SCFA (acetic, propionic, and butyric acids; 40.08, 15.41, and 9.78 mM respectively). a, b, means without a common superscript differ (*P* < 0.05)

**Fig. 7:**
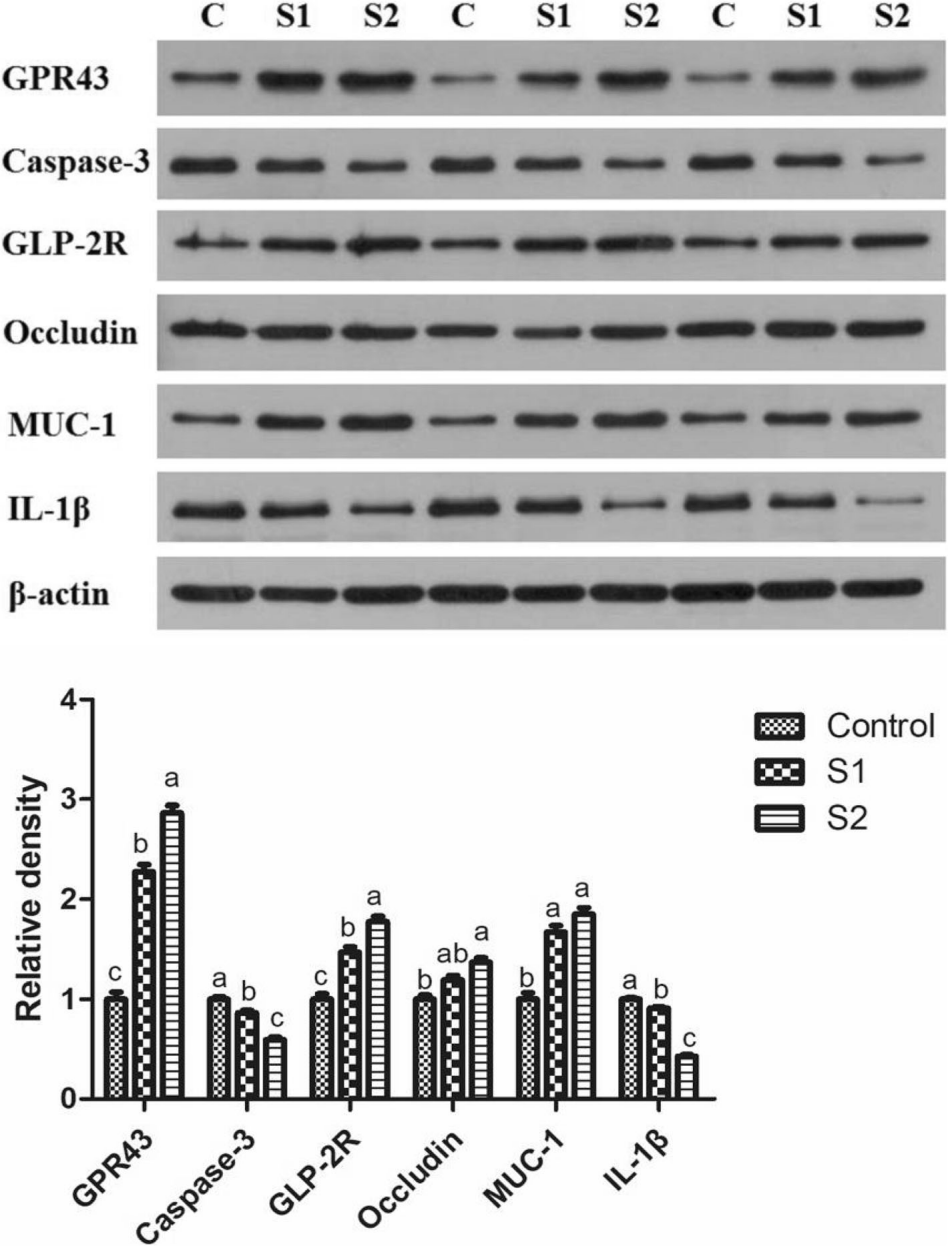
Effect of gastric infusion of SCFA on the protein levels of GPR43, Caspase-3, GLP-2, Occludin, MUC-1, and IL-1β in jejunum of weaned piglets. S1, pigs treated with SCFA (acetic, propionic, and butyric acids; 20.04, 7.71, and 4.89 mM respectively); S2, pigs treated with SCFA (acetic, propionic, and butyric acids; 40.08, 15.41, and 9.78 mM respectively). a, b, means without a common superscript differ (*P* < 0.05)

**Fig. 8:**
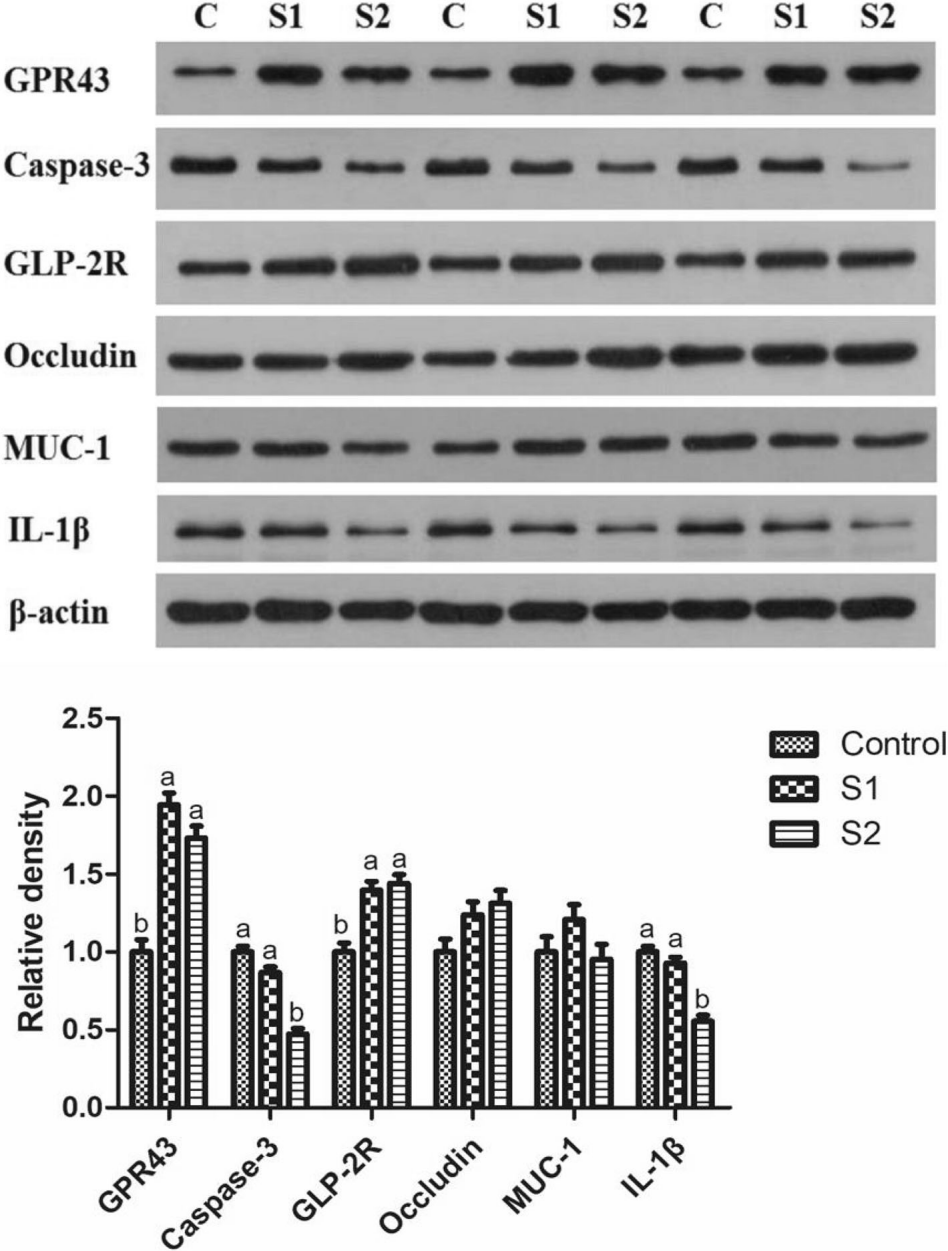
Effect of gastric infusion of SCFA on the protein levels of GPR43, Caspase-3, GLP-2, Occludin, MUC-1, and IL-1β in colon of weaned piglets. S1, pigs treated with SCFA (acetic, propionic, and butyric acids; 20.04, 7.71, and 4.89 mM respectively); S2, pigs treated with SCFA (acetic, propionic, and butyric acids; 40.08, 15.41, and 9.78 mM respectively). a, b, means without a common superscript differ (*P* < 0.05)

Gastric infusion of high concentration of SCFA increased the T-AOC activity of the jejunum (Table 6, *P* = 0.075) and decreased the contents of MDA in the jejunum and colon relative to the control group (*P* < 0.05). Moreover, compared with the control group, gastric infusion of SCFA increased the relative mRNA expressions of Claudin-1 in the jejunum, Occludin, and Claudin-1 in the duodenum and ileum (Fig. 4, *P* < 0.05). Pigs of S2 group had higher relative mRNA expression of Occludin in the jejunum than pigs of control group (*P* < 0.05), and pigs of S1 group had higher relative mRNA expression of Claudin-1 in the colon than pigs of control group (*P* < 0.05). Meanwhile, compared with the control group, gastric infusion of SCFA increased relative MUC1 mRNA expressions in the jejunum and the number of goblet cells in the ileum and colon (Fig. 5 and Table 3, *P* < 0.05), while S1 group had higher relative mRNA expressions of MUC1 and MUC2 in the ileum (*P* < 0.05). Besides, gastric SCFA infusion enhanced the counts of *Lactobacillus* spp in the ileal digesta and decreased the numbers of *Escherichia coli* in the ileal digesta of pigs (Table 7, *P* < 0.05). An increase in the *Lactobacillus* spp populations of cecal digesta was found in S2 group compared with control group (*P* < 0.05), while a decreasing number of *Escherichia coli* of cecal and colonic digesta was found in S2 group compared with control group (*P* < 0.05). In addition, gastric infusion of SCFA decreased the relative mRNA expressions of IL-10 in the jejunum, IL-1β in the ileum, and IL-1β and IL-8 in the colon (Fig. 6, *P* < 0.05). The relative mRNA expressions of IL-1β in the duodenum and jejunum, and IL-8 in the jejunum and ileum in the S2 group were lower than those in the control group. Finally, as shown in Figs. 7 and 8, the protein levels of Occludin and MUC1 in the jejunum of pigs in S2 group were higher than those in the control group, whereas the protein level of IL-1β in the colon of pigs in S2 group was lower than that in the control group (*P* < 0.05). The pigs of S1 and S2 groups had lower protein level of IL-1β in the jejunum than pigs of control group, and the pigs of S2 group had lower protein level of IL-1β in the jejunum than pigs of S1 group (*P* < 0.05).

**Table 6:** Effect of gastric infusion of SCFA on antioxidant capacity of jejunum and colon in weaned piglets

Items	Control	S1	S2	SEM	*P* value
Jejunum					
T-AOC (U/mgprot)	0.345^a^	0.391^ab^	0.495^a^	0.042	0.075
MDA (nmol/mgprot)	1.005^a^	0.860^ab^	0.661^b^	0.083	0.045
Colon					
T-AOC (U/mgprot)	0.268	0.337	0.347	0.028	0.138
MDA (nmol/mgprot)	0.914^a^	0.607^ab^	0.315^b^	0.124	0.021

S1, pigs treated with SCFA (acetic, propionic, and butyric acids; 20.04, 7.71, and 4.89 mM respectively); S2, pigs treated with SCFA (acetic, propionic, and butyric acids; 40.08, 15.41, and 9.78 mM respectively)

^a-b^Within a row, means without a common superscript differ (*P* < 0.05)

**Table 7:** Effect of gastric infusion of SCFA on the ileal, caecal, and colonic *Escherichia coli*, *Lactobacillus* spp, *Bifidobacterium* spp, and *Bacillus* spp in weaned piglets (log copies/g)

Items	Control	S1	S2	SEM	*P* value
Ileum					
Total bacteria	10.550^a^	10.103^ab^	9.962^b^	0.137	0.031
*Bacillus* spp	8.487	8.495	8.480	0.168	0.999
*Lactobacillus* spp	7.043^b^	7.784^a^	8.231^a^	0.185	0.004
*Escherichia coli*	9.957^a^	8.780^b^	8.713^b^	0.277	0.016
*Bifidobacterium* spp	7.686	7.939	8.202	0.242	0.354
Cecum					
Total bacteria	11.672^a^	11.451^ab^	11.336^b^	0.063	0.011
*Bacillus* spp	9.400	9.332	9.274	0.186	0.894
*Lactobacillus* spp	8.037^b^	8.506^ab^	8.900^a^	0.160	0.011
*Escherichia coli*	10.182	8.922	9.203	0.344	0.063
*Bifidobacterium* spp	7.940	8.163	8.241	0.206	0.579
Colon					
Total bacteria	11.473	11.444	11.372	0.152	0.888
*Bacillus* spp	9.568	9.362	9.332	0.090	0.182
*Lactobacillus* spp	8.651	9.065	8.899	0.164	0.244
*Escherichia coli*	10.009^a^	9.103^ab^	8.952^b^	0.262	0.036
*Bifidobacterium* spp	7.643	7.844	7.886	0.101	0.241

S1, pigs treated with SCFA (acetic, propionic, and butyric acids; 20.04, 7.71, and 4.89 mM respectively); S2, pigs treated with SCFA (acetic, propionic, and butyric acids; 40.08, 15.41, and 9.78 mM respectively)

^a, b^Within a row, means without a common superscript differ (*P* < 0.05)

## Discussion

Some reports have stated that excessive SCFA are harmful and could damage the intestinal function [5, 25], so further research is needed to verify the effects of different SCFA concentrations with the same ratio on the gut development. Meanwhile, piglets have increasing stress after weaning. Based on the above considerations, our study was used to investigate whether gastric infusion of different concentrations of SCFA could attenuate the negative effects of weaning in the perspective of actual production.

Previous studies on pigs and lambs have indicated that dietary supplementation with 0.17% or 1.25% sodium butyrate significantly increased the content of butyric acid in the serum [18, 26]. In the present study, gastric infusion of SCFA increased the concentration of butyric acid in the serum, the concentrations of acetic acid, propionic acid, butyric acid, and total SCFA in the ileal, cecal, or colonic digesta, which were accompanied with higher relative mRNA expression of GPR41 in the colon and GPR43 in ileum and colon. Moreover, we found the contents of acetic acid, propionic acid, butyric acid, and total SCFA in the serum; acetic acid, propionic acid, and total SCFA in the ileal digesta; and the butyric acid and total SCFA in the colonic digesta in high-concentration infusion group were higher than those in low-concentration infusion group. Therefore, the SCFA could arrive in the hindgut of piglets through gastric infusion.

Interestingly, the in vitro studies disclosed that SCFA or butyrate suppressed epithelial proliferation, decreased cell viability, and induced apoptosis [7, 10, 27], while the in vivo result was different with the in vitro studies. A previous study showed that 10 mM butyric acid incubation of human colonic biopsies for 3 h resulted in the increasing proportion of proliferating cells per crypt [28]. Studies using infusion model on rats and pigs also suggested that infusion of SCFA (acetic, propionic, and butyric acids are 75, 35, and 20 mM, respectively) into the ileum, cecum, or colon could stimulate cell proliferation in the small intestine, cecum, and colon, and this effect was dose-dependent of SCFA but independent of the presence of gut bacteria [7, 9, 29]. The proliferative effect was also observed in our study, which found the percentage of jejunal and colonic apoptotic cells, the pro-apoptosis gene (Bax and Caspase-3) abundances, and the pro-apoptosis protein (Caspase-3) level were decreased in SCFA infusion groups. Moreover, the proportion of jejunal G0G1 phase cells in high-concentration infusion group was lower than that in low-concentration infusion group, which indicated the stimulative proliferation effect was dose-dependent of SCFA within certain concentration. Therefore, SCFA have paradoxical effects on cell proliferation and apoptosis in in vivo and in vitro studies, as they show stimulative proliferation effect in in vivo of normal gut [30, 31], whereas display inhibitive proliferation effect in tumor cells owing to the Warburg effect, which makes SCFA function as HDAC inhibitors, and thereby inhibit proliferation [32].

Weaning is the most severe stress after pigs are born, which is usually characterized by the decrease of villus height and the increase of crypt depth in the small intestine, in association with a reduction in the nutrient digestibility [33, 34]. Therefore, for digesting and utilizing nutrients after weaning, maintaining the intestinal morphological and structural properties is important. In the present study, gastric infusion of SCFA increased the villus height of jejunum and ileum, and villus height:crypt depth of duodenum and jejunum in piglets. Based on the foregoing view, we showed that gastric infusion of SCFA could modulate intestinal morphological changes and, consequently, enhance nutrient absorption and be beneficial to intestinal growth and development.

In general, morphological changes in the intestinal tissue were accompanied by alteration of DNA and protein concentrations [35]. By examining the intestinal DNA and protein concentrations, we confirmed that gastric infusion of SCFA increased the DNA concentration of jejunal mucosa and protein concentrations of duodenal and jejunal mucosa, especially in the high-dose infusion group. Meanwhile, we also verified the increases in the relative mRNA expressions of intestinal development-related genes, including IGF-1, IGF-IR, GLP-2, and GLP-2R, which indicated gastric infusion of SCFA could be beneficial to the gut morphology and development. Consistent with our result, intracolonic infusion of SCFA (acetic, propionic, and butyric acids are 75, 35, and 20 mM, respectively) for 7 days increased the contents of mucosal protein, RNA, and DNA, as well as mucosal weight of colon in rats [8], and dietary 1000 or 1700 mg/kg sodium butyrate supplementation increased villus height and villus height:crypt depth in the intestine of pigs [17, 18]. As is known to us, GLP-2 is a specific growth factor of intestinal epithelial cell and plays an important role in intestinal development, digestion and absorption, blood flow, and integrity of intestine [36]. In our study, high dose of SCFA infusion increased ileal GLP-2 concentration and GLP-2 receptor level, suggesting SCFA may promote intestinal development through elevating GLP-2 secretion.

The integrity of intestinal barrier function is closely related to intestinal health, which also plays an important role in gut development, and thus improves the digestion and absorption ability [37]. Generally, the intestinal redox state affects the intestinal epithelial integrity. Here, we reported that high dose of SCFA infusion decreased the MDA concentration and increased SOD activity in jejunum. In accordance with our result, exposing vascular smooth muscle cell to 5 mM butyrate for 48 h upregulated the GPx-3 and GPx-4 mRNA abundances [23]. Also, it is well recognized that tight junction between epithelial cells is one of the main components of the intestinal physical barrier between epithelial cells, and monitoring the tight junction proteins can be used to evaluate intestinal permeability and epithelial integrity [38]. In physiological concentrations, sodium butyrate (4 mM, 48 h) promoted intestinal epithelial integrity as measured through increasing the relative mRNA expression of tight junction (Occludin and ZO-1) in IPEC-J2 cells [13]. In our study, SCFA infusion increased the relative mRNA expressions of Claudin-1 in the jejunum, and Occludin and Claudin-1 in the duodenum and ileum. Similarly, the protein level of Occludin in the jejunum of pigs in high-SCFA concentration infusion group was higher than that in the control group, which suggested that SCFA could alleviate the weaning-induced damage to intestinal structural integrity by promoting the tight junction protein expression levels in weaned pigs.

Besides physical barrier, the maintenance of chemical barrier and immune barrier function is crucial for gut health. The study in a pig model revealed that dietary 500 mg/kg sodium butyrate supplementation reduced the pro-inflammatory cytokine (TNF-a and IL-6) levels in the serum [17]. In an in vitro study, demonstrated SCFA (1 to 5 mM Butyrate, 1 to 5 mM propionate or 20 mM acetate) could downregulate IL-8 and IL-1β mRNA abundances in Caco-2 cells induced by LPS [16]. Certain anti-inflammatory effects of SCFA on gut function may, to some extent, occur through two SCFA receptors (GPR41 and GPR43) [39]. Similarly, in our study, gastric infusion of SCFA decreased IL-1β and/or IL-8 mRNA abundance in the ileum and colon, increased mRNA expression of GPR41 and/or GPR43 in the jejunum, ileum, and colon, and elevated the GPR43 protein level in the jejunum and colon, especially in high-dose infusion group. Usually, mucins secreted by goblet cells in the gut seem to contribute to a mucus layer and provide a chemical barrier to the intestine [40]. A previous study has shown that there was a positive correlation between the total thickness of the mucous layer and colonic SCFA concentrations [41]. Accordingly, in our study, gastric infusion of SCFA increased the relative mRNA expression and protein level of MUC1 in the jejunum and enhanced the numbers of goblet cells in the ileum and colon, which was generally consistent with studies using in vitro models. In these in vitro researches, SCFA (5 to 15 mM acetic, propionic, or butyric acids) stimulate MUC2 expression through MAPK signaling pathway, leading to a better intestinal epithelial chemical barrier [15, 42, 43]. It appears, therefore, that SCFA infusion could maintain intestinal chemical and immune barrier by regulating the productions of mucins and inflammatory cytokines.

Intestinal microbiota is an integral part of gut health, which constitutes the intestinal biological barriers. The alteration and balance between harmful bacteria (pathogenic *Escherichia coli*) and beneficial bacteria (*Lactobacillus* spp and *Bifidobacterium* spp) in the gut are associated with the intestinal morphology [44]. Greater SCFA productions have been reported to decrease the number of potential pathogens (such as *Escherichia coli* and *Salmonella*) in pigs [45]. Our results demonstrated that gastric infusion of SCFA enhanced the *Lactobacillus* spp populations in ileal and cecal digesta, and decreased the *Escherichia coli* populations in ileal, cecal, and colonic digesta. One potential explanation is that SCFA may decrease pH values of digesta provide an acidic environment for more beneficial bacteria to exist, further competitively exclude harmful bacteria and sustain the gut microecosystem [46]. An in vitro study found that increasing the butyrate concentration from 0 to 9 mM reduced the adherent abilities of *Escherichia coli* as well as increased adherence of *Lactobacillus acidophilus* and *Bifidobacterium longum* [15]. Also, some in vivo studies get the similar results and reveal that dietary supplemented with 1000 or 1700 mg/kg sodium butyrate changed the composition of microbiota [17, 18]. Furthermore, *Escherichia coli* has been reported to destabilize and dissociate tight junction proteins [47]. In the current study, the increased Occludin and Claudin-1 expression levels in the SCFA infusion group were in accordance with the decreased *Escherichia coli* populations. These combined findings suggested that SCFA could decrease pH values, maintain the balance of gut microbiota, prevent tight junction proteins from dissociating, and thus improve intestinal barrier.

## Conclusions

**Fig. 9:**
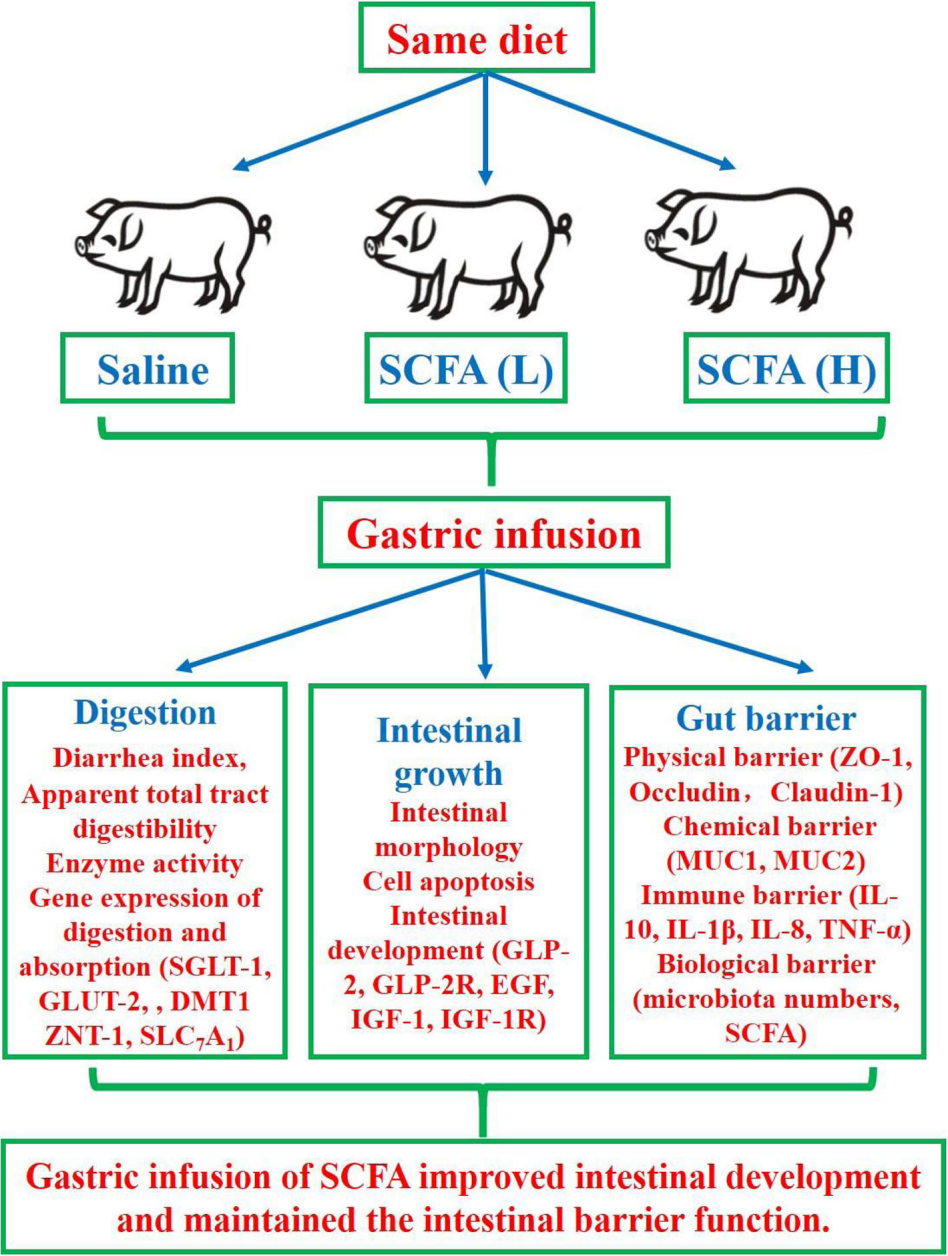
The overall frame diagram. SCFAs (L), pigs treated with SCFA (acetic, propionic, and butyric acids; 20.04, 7.71, and 4.89 mM respectively); S2, pigs treated with SCFA (acetic, propionic, and butyric acids; 40.08, 15.41, and 9.78 mM respectively)

In summary, gastric infusion of SCFA, especially high concentration of SCFA, increased SCFA concentrations in the serum and intestine, decreased apoptosis of epithelial cells, stimulated intestinal DNA and protein concentrations, and improved gut barrier function in weaned pigs (Fig. 9).

## Materials and methods

### Animals, management, diets, and sample collection

All experimental procedures and animal care were accomplished in accordance with the Guide for the Care and Use of Laboratory Animals provided by the Institutional Animal Care Advisory Committee for Sichuan Agricultural University. All animal protocols used in this study were approved by the Animal Care and Use Committee of Sichuan Agricultural University under permit number DKY-B20131704.

A total of 21 weaned DLY barrows (Duroc × Landrance × Yorkshire) provided by a commercial pig farm with an average initial body weight of 8.31 ± 0.72 kg (24 days of age) were randomly allotted into 3 treatments (*n* = 7). The treatment groups were (1) control; (2) low SCFA concentration (acetic, propionic, and butyric acids are 20.04, 7.71, and 4.89 mM respectively), S1; (3) high SCFA concentration (acetic, propionic, and butyric acids are 40.08, 15.41, and 9.78 mM respectively), S2. Before feeding, piglets in each treatment were intragastrically infused with either 200 mL SCFA (S1 and S2 groups) or sterile saline (control group) in the morning of each day. The SCFA solutions were obtained by adding analytically pure SCFA into sterile saline. The experimental period lasted for 7 days.

All pigs were individually housed in metabolism cages (1.0 × 0.5 × 0.8 m) with a self-feeder and a nipple watering device in a temperature (25 ± 1 °C), humidity (60 ± 5%), and light-controlled room during the study. The pigs had ad libitum access to feeding and drinking water. Diet was formulated to meet or exceed the nutrient recommendation of NRC (2012) for 7–11 kg pigs (Additional file 1: Table S1). The condition of each piglet was monitored at least on a daily basis by manual and visual inspections.

On day 8, all pigs were anesthetized with an intravenous injection of Zoletil 50 (Beijing PET Technology Co., LTD, Beijing, China, 10 mg/kg BW), and the blood samples were collected from the portal vein into sterile vacuum tubes and centrifuged at 3500 rmp for 10 min. After blooding, the pigs were killed by anesthesia and jugular exsanguinations. The length of small intestine and large intestine was measured, and the tissues of duodenum, jejunum, ileum, and colon (approximately 2 cm in length) were immediately isolated and stored in 4% paraformaldehyde solution. Then, the tissues of ileum and colon were isolated and preserved in phosphate buffer solution (4 °C). This was followed by collecting the digesta of stomach, duodenum, jejunum, ileum, caecum, and colon, and each pH value was measured with a pH meter (PHS-3C pH, Shanghai, China). After that, the weight of small intestine and large intestine was detected and recorded. Besides, the tissues and mucosa of duodenum, jejunum, ileum, and colon were immediately collected and stored at − 80 °C. The intestinal index was carried out by the formulas as described by Diao et al. (2016) [48]. Relative density of intestine (g/cm) = intestinal weight/intestinal length × 100, relative length of intestine (cm/g) = intestinal length/body weight, and relative weight of intestine (%) = intestinal weight/body weight × 100.

### Short-chain fatty acid

Acetic acid, propionic acid, butyric acid, and total SCFA were separated and quantified from serum and digesta of ileum, cecum, and colon in a gas chromatographic system (VARIAN CP-3800, Varian, Palo Alto, CA, USA) as previously described by Franklin et al. (2002) [49].

### Histology of intestine

As described by Touchette et al. (2002) [50], the duodenum, jejunum, ileum, and colon were douched with sterile saline and stored in 4% paraformaldehyde solution, and then were dehydrated and embedded in paraffin wax. The preserved samples were prepared after installing and staining with hematoxylin and eosin. Ten intact and well-orientated sections (villi height and their adjoined crypts) of each sample were determined with an image processing and analysis system (Image-Pro Plus 4.5, Silver Spring, MD, USA) at × 40 magnification. The number of goblet cells was measured using alcian blue and periodic acid schiff (AB-PAS) [51].

### Apoptosis and cell cycle of ileal and colonic epithelial cell by flow cytometry

The epithelial cells of jejunum and colon were isolated to measure the proportion of apoptotic cells and cell cycle by flow cytometry as described before [52]. Briefly, the mucosal layer of jejunum and colon was isolated, and then cell suspension was formed by grinding and filtering. This was followed by modulating cell concentration to 1 × 10^6^ cells/ml. After that, 5 μl Annexin V-fluorescein isothiocyanate (V-FITC) and 5 μl propidium iodide (PI) were added into 100 μl cell suspension, and incubated for 15 min (room temperature), and then added 400 μl 1 × binding buffer. The apoptotic cells were carried out using Cell Quest software within 1 h. Similarly, 1 ml 0.25% Tritonx-100 was added into 100 μl cell suspension. Then, cells were incubated for 30 min (4 °C), centrifuged at 1000 rmp for 5 min, discarded the supernatant, and added 5 μl PI and 500 μl pre-cooling PBS one after another. The cell cycle was measured using Modifit software within 1 h.

### Total protein, antioxidant capacity, DNA, and GLP-2 concentration

The mucosa of duodenum, jejunum, ileum, and colon was homogenized after dilution with sterile saline (*m*:*v* = 1:9), and then centrifuged at 2500 rpm for 15 min. The total protein concentrations of intestinal mucosa samples were measured by the Bradford brilliant blue method.

The antioxidant capacity (methane dicarboxylic aldehyde (MDA) and total antioxidant capacity (T-AOC)) was determined using commercial kits (Nanjing Jiancheng Institute of Bioengineering, Jiangsu, China).

The glucagon-like peptide 2 (GLP-2) concentration of the jejunal and colonic mucosa was determined using swine ELISA kits (R&D Systems China Company Limited, Minneapolis, MN, USA), and quantified using a BioTek Synergy HT microplate reader (BioTek Instruments, Winooski, VT, USA).

The genomic DNA was isolated from the frozen mucosa of duodenum, jejunum, ileum, and colon with a TIANamp genomic DNA kit (TIANGEN, Beijing, China) according to the manufacturer’s protocols. The concentration of total DNA was analyzed spectrophotometrically using a Beckman Coulter DU 800 (Beckman Coulter).

### Enumeration of *Escherichia coli*, *Lactobacillus* spp, *Bifidobacterium* spp, *Bacillus* spp, and total bacteria by PCR

Bacterial DNA was extracted from digesta of ileum, cecum, and colon using Stool DNA Kits (Omega Bio-tek, Doraville, CA, USA). The primers and fluorescent oligonucleotide probes (Additional file 1: Table S2) for total bacteria, *Escherichia coli*, *Lactobacillus* spp, *Bifidobacterium* spp, and *Bacillus* spp were obtained from the published papers [53, 54], which were synthesized by Invitrogen (Shanghai, China). The PCR conditions, reaction system, and calculation method were referring to Qi, et al. (2011) [53]. These special kinds of bacteria can be detected by the CFX96 Real-Time PCR Detection System (Bio-Rad, CA, USA).

### Total RNA extraction, reverse transcription reaction, and real-time quantitative PCR

Total RNA was isolated from the frozen mucosa of duodenum, jejunum, ileum, and colon using the TRIzol reagent (Takara, Dalian, China) according to the manufacturer’s protocols. The cDNA of each sample was acquired by reverse transcription with a PrimeScript RT reagent kit (Takara, Dalian, China). The genes related to SCFA receptors (GPR41, G protein-coupled receptor-41; GPR43, G protein-coupled receptor-43), intestinal development (GLP-2; GLP-2R, glucagon-like peptide-2 receptor; EGF, epidermal growth factor; IGF-1, insulin-like growth factor-1; IGF-1R, insulin-like growth factor-1 receptor), cell apoptosis and cycle (Caspase-3, cysteinyl aspartate-specific proteinase-3; Bcl-2, B cell lymphoma/lewkmia-2; Bax, Bcl-2-associated X protein; Cyclin D1; p21/Cip1, cyclin-dependent kinase inhibitor 1A), and intestinal barrier (Occludin; Claudin-1; ZO-1, zonula occludens 1; MUC1, mucin 1; MUC2, mucin 2; IL-10, interleukin-10; IL-1β, interleukin-1β; IL-8, interleukin-8; TNF-α, tumor necrosis factor α) can be detected by the QuantStudio™ Real-Time PCR System (Thermo Fisher Scientific, Shanghai, China) as described by Chen et al. (2013) [37]. The primers (Additional file 1: Table S3) were synthesized by Invitrogen (Shanghai, China).

### Analysis of protein levels by western blot

The antibodies against GPR43, Caspase-3, GLP-2, Occludin, MUC-1, IL-1β, and β-actin were purchased from Abcam (Cambridge, MA, USA), Cell Signaling Technology (Davers, MA), and Santa Cruz Biotechnology Inc. (Santa Cruz, CA, USA), respectively. Protein levels for the GPR43, Caspase-3, GLP-2, Occludin, MUC-1, IL-1β, and β-actin in jejunal and colonic mucosa were determined by western blot analysis as described previously [55].

### Statistical analysis

In this study, each pig was used as the statistical unit. All the data were checked for normal distribution and homogeneity of variance using Shapiro-Wilk and Levene’s tests, respectively, in SAS 9.1 (SAS Inst. Inc., NC, USA). When the data were recognized as normally distributed and exhibited homogeneity of variance, data were analyzed by one-way ANOVA and Duncan’s multiple comparison. All differences were considered significant at *P* < 0.05, whereas *P* values between 0.05 and 0.10 were considered a trend. All results were expressed as mean and SEM.

## Additional file

Additional file 1: **Table S1.** Composition and nutrient level of experimental diets. **Table S2.** Primers and probes for real-time PCR of bacteria. Table S3. Primer sequences and annealing temperature of pigs. (DOCX 30 kb)

## References

[1] Knudsen KEB, Hedemann MS, Lærke HN (2012). The role of carbohydrates in intestinal health of pigs. Anim Feed Sci Tech.

[2] Louis P, Scott KP, Duncan SH, Flint HJ (2007). Understanding the effects of diet on bacterial metabolism in the large intestine. J Appl Microbiol.

[3] Bugaut M (1987). Occurrence, absorption and metabolism of short chain fatty acids in the digestive tract of mammals. Comp Biochem Physiol B.

[4] Louis P, Flint HJ (2009). Diversity, metabolism and microbial ecology of butyrate-producing bacteria from the human large intestine. FEMS Microbiol Lett.

[5] Lin J (2013). Effects of short chain fatty acids on the intestinal barrier. Curr Nutr Food Sci.

[6] Scheppach W (1994). Effects of short chain fatty acids on gut morphology and function. Gut.

[7] Sakata T (1987). Stimulatory effect of short-chain fatty acids on epithelial cell proliferation in the rat intestine: a possible explanation for trophic effects of fermentable fibre, gut microbes and luminal trophic factors. Br J Nutr.

[8] Kripke SA, Fox AD, Berman JM, Settle RG, Rombeau JL (1989). Stimulation of intestinal mucosal growth with intracolonic infusion of short-chain fatty acids. JPEN J Parenter Enteral Nutr.

[9] Kien CL, Blauwiekel R, Bunn JY, Jetton TL, Frankel WL, Holst JJ (2007). Cecal infusion of butyrate increases intestinal cell proliferation in piglets. J Nutr.

[10] Peng L, He Z, Chen W, Holzman IR, Lin J (2007). Effects of butyrate on intestinal barrier function in a Caco-2 cell monolayer model of intestinal barrier. Pediatr Res.

[11] Turner JR (2009). Intestinal mucosal barrier function in health and disease. Nat Rev Immunol.

[12] Alva-Murillo N, Ochoa-Zarzosa A, Lopez-Meza JE (2012). Short chain fatty acids (propionic and hexanoic) decrease Staphylococcus aureus internalization into bovine mammary epithelial cells and modulate antimicrobial peptide expression. Vet Microbiol.

[13] Ma X, Fan PX, Li LS, Qiao SY, Zhang GL, Li DF (2012). Butyrate promotes the recovering of intestinal wound healing through its positive effect on the tight junctions. J Anim Sci.

[14] Kelly CJ, Zheng L, Campbell EL, Saeedi B, Scholz CC, Bayless AJ, Wilson KE, Glover LE, Kominsky DJ, Magnuson A (2015). Crosstalk between microbiota-derived short-chain fatty acids and intestinal epithelial HIF augments tissue barrier function. Cell Host Microbe.

[15] Jung TH, Park JH, Jeon WM, Han KS (2015). Butyrate modulates bacterial adherence on LS174T human colorectal cells by stimulating mucin secretion and MAPK signaling pathway. Nutr Res Pract.

[16] Iraporda C, Errea A, Romanin DE, Cayet D, Pereyra E, Pignataro O, Sirard JC, Garrote GL, Abraham AG, Rumbo M (2015). Lactate and short chain fatty acids produced by microbial fermentation downregulate proinflammatory responses in intestinal epithelial cells and myeloid cells. Immunobiology.

[17] Wen ZS, Lu JJ, Zou XT (2012). Effects of sodium butyrate on the intestinal morphology and DNA-binding activity of intestinal nuclear factor-κB in weanling pigs. J Anim Vet Adv.

[18] Galfi P, Bokori J (1990). Feeding trial in pigs with a diet containing sodium n-butyrate. Acta Vet Hung.

[19] Aruoma OI, Grootveld M, Bahorun T (2006). Free radicals in biology and medicine: from inflammation to biotechnology. Biofactors.

[20] Yin J, Ren W, Liu G, Duan J, Yang G, Wu L, Li T, Yin Y (2013). Birth oxidative stress and the development of an antioxidant system in newborn piglets. Free Radic Res.

[21] Lallès JP, Boudry G, Favier C, Floc'h NL, Huêrou-Luron IL, Montagne L, Oswald IP, Pié S, Piel C, Sève B (2004). Gut function and dysfunction in young pigs: physiology. Physiology.

[22] Jacob RA (1995). The integrated antioxidant system. Nutr Res.

[23] Ranganna K, Mathew O, Milton S (2014). Link between antioxidant effect and antiinflammatory response in butyrate arrested vascular smooth muscle cells (VSMC) proliferation (LB250). FASEB J.

[24] Pluske JR, Hampson DJ, Williams IH (1997). Factors influencing the structure and function of the small intestine in the weaned pig: a review. Livest Prod Sci.

[25] Ploger S, Stumpff F, Penner GB, Schulzke JD, Gabel G, Martens H, Shen Z, Gunzel D, Aschenbach JR (2012). Microbial butyrate and its role for barrier function in the gastrointestinal tract. Ann N Y Acad Sci.

[26] Wilson DJ, Mutsvangwa T, Penner GB (2012). Supplemental butyrate does not enhance the absorptive or barrier functions of the isolated ovine ruminal epithelia. J Anim Sci.

[27] Clarke JM, Young GP, Topping DL, Bird AR, Cobiac L, Scherer BL, Winkler JG, Lockett TJ (2012). Butyrate delivered by butyrylated starch increases distal colonic epithelial apoptosis in carcinogen-treated rats. Carcinogenesis.

[28] Bartram HP, Scheppach W, Schmid H, Hofmann A, Dusel G, Richter F, Richter A, Kasper H (1993). Proliferation of human colonic mucosa as an intermediate biomarker of carcinogenesis: effects of butyrate, deoxycholate, calcium, ammonia, and pH. Cancer Res.

[29] Sakata T, von Engelhardt W (1983). Stimulatory effect of short chain fatty acids on the epithelial cell proliferation in rat large intestine. Comp Biochem Physiol A Comp Physiol.

[30] Sengupta S, Muir JG, Gibson PR (2006). Does butyrate protect from colorectal cancer?. J Gastroen Hepatol.

[31] Lupton JR (1995). Butyrate and colonic cytokinetics: differences between in vitro and in vivo studies. Eur J Cancer Prev.

[32] Donohoe DR, Collins LB, Wali A, Bigler R, Sun W, Bultman SJ (2012). The Warburg effect dictates the mechanism of butyrate-mediated histone acetylation and cell proliferation. Mol Cell.

[33] Montagne L, Pluske JR, Hampson DJ (2003). A review of interactions between dietary fibre and the intestinal mucosa, and their consequences on digestive health in young non-ruminant animals. Anim Feed Sci Tech.

[34] Wang Y, Shan T, Xu Z, Liu J, Feng J (2006). Effect of lactoferrin on the growth performance, intestinal morphology, and expression of PR-39 and protegrin-1 genes in weaned piglets. J Anim Sci.

[35] Hu Z, Guo Y (2007). Effects of dietary sodium butyrate supplementation on the intestinal morphological structure, absorptive function and gut flora in chickens. Anim Feed Sci Tech.

[36] Bloom SR, Polak JM (1982). The hormonal pattern of intestinal adaptation. A major role for enteroglucagon. Scand J Gastroenterol Suppl.

[37] Chen H, Mao X, He J, Yu B, Huang Z, Yu J, Zheng P, Chen D (2013). Dietary fibre affects intestinal mucosal barrier function and regulates intestinal bacteria in weaning piglets. Br J Nutr.

[38] Heiskala M, Peterson PA, Yang Y (2001). The roles of claudin superfamily proteins in paracellular transport. Traffic.

[39] Le Poul E, Loison C, Struyf S, Springael JY, Lannoy V, Decobecq ME, Brezillon S, Dupriez V, Vassart G, Van Damme J, Parmentier M, Detheux M (2003). Functional characterization of human receptors for short chain fatty acids and their role in polymorphonuclear cell activation. J Biol Chem.

[40] Lamont JT (1992). Mucus: the front line of intestinal mucosal defense. Ann N Y Acad Sci.

[41] Hedemann MS, Theil PK, Bach Knudsen KE (2009). The thickness of the intestinal mucous layer in the colon of rats fed various sources of non-digestible carbohydrates is positively correlated with the pool of SCFA but negatively correlated with the proportion of butyric acid in digesta. Br J Nutr.

[42] Willemsen LE, Koetsier MA, van Deventer SJ, van Tol EA (2003). Short chain fatty acids stimulate epithelial mucin 2 expression through differential effects on prostaglandin E(1) and E(2) production by intestinal myofibroblasts. Gut.

[43] Burger-van Paassen N, Vincent A, Puiman PJ, van der Sluis M, Bouma J, Boehm G, van Goudoever JB, van Seuningen I, Renes IB (2009). The regulation of intestinal mucin MUC2 expression by short-chain fatty acids: implications for epithelial protection. Biochem J.

[44] Diao H, Zheng P, Yu B, He J, Mao X, Yu J, Chen D (2015). Effects of benzoic acid and thymol on growth performance and gut characteristics of weaned piglets. Asian Austral J Anim Sci.

[45] Prohászka L, Jayarao BM, Fábián A, Kovács S (1990). The role of intestinal volatile fatty acids in the *Salmonella* shedding of pigs. J Veterinary Med Ser B.

[46] Duncan SH, Louis P, Thomson JM, Flint HJ (2009). The role of pH in determining the species composition of the human colonic microbiota. Environ Microbiol.

[47] Muza-Moons MM, Schneeberger EE, Hecht GA (2004). Enteropathogenic *Escherichia coli* infection leads to appearance of aberrant tight junctions strands in the lateral membrane of intestinal epithelial cells. Cell Microbiol.

[48] Diao H, Yan HL, Xiao Y, Yu B, Yu J, He J, Zheng P, Zeng BH, Wei H, Mao XB (2016). Intestinal microbiota could transfer host gut characteristics from pigs to mice. BMC Microbiol.

[49] Franklin MA, Mathew AG, Vickers JR, Clift RA (2002). Characterization of microbial populations and volatile fatty acid concentrations in the jejunum, ileum, and cecum of pigs weaned at 17 vs 24 days of age. J Anim Sci.

[50] Touchette KJ, Carroll JA, Allee GL, Matteri RL, Dyer CJ, Beausang LA, Zannelli ME (2002). Effect of spray-dried plasma and lipopolysaccharide exposure on weaned pigs: I. Effects on the immune axis of weaned pigs. J Anim Sci.

[51] Kunert KS, Tisdale AS, Gipson IK (2002). Goblet cell numbers and epithelial proliferation in the conjunctiva of patients with dry eye syndrome treated with cyclosporine. Arch Ophthalmol.

[52] Tao C, Hengmin C, Yun C, Caimin B, Tao G, Xi P (2011). Cell-cycle blockage associated with increased apoptotic cells in the thymus of chickens fed on diets high in fluorine. Hum Exp Toxicol.

[53] Qi H, Xiang Z, Han G, Yu B, Huang Z, Chen D (2011). Effects of different dietary protein sources on cecal microflora in rats. Afr J Biotechnol.

[54] Fierer N, Jackson JA, Vilgalys R, Jackson RB (2005). Assessment of soil microbial community structure by use of taxon-specific quantitative PCR assays. Appl Environ Microbiol.

[55] Suryawan A, Nguyen HV, Bush JA, Davis TA (2001). Developmental changes in the feeding-induced activation of the insulin-signaling pathway in neonatal pigs. Am J Physiol Endocrinol Metab.

