# Supplementary material for: Gastric infusion of short-chain fatty acids can improve intestinal barrier function in weaned piglets
Source: Genes Nutr. 2019 Feb 1;14:4. doi: 10.1186/s12263-019-0626-x (PMC6359775; doi:10.1186/s12263-019-0626-x)
Supplement: Supplementary file 1 — Table S1. Composition and nutrient level of experimental diets. Table S2. Primers and probes for real-time PCR of bacteria. Table S3. Primer sequences and annealing temperature of pigs. (DOCX 30 kb) [file 12263_2019_626_MOESM1_ESM.docx]

Table S1 Composition and nutrient level of experimental diets (air dry basis %)

| Ingredient | Content | Calculated Composition | Nutrient content |
| --- | --- | --- | --- |
| Corn | 27.79 | DE (MJ/kg) | 3.55 |
| Extruded corn | 28.61 | Crude protein | 19.59 |
| Dehulled soybean meal | 10.33 | Calcium | 0.81 |
| Extruded soybean | 4.50 | Total phosphorus | 0.57 |
| Fish meal | 0.50 | Available phosphorus | 0.37 |
| Whey powder | 8.00 | Lys | 1.36 |
| Soybean protein concentrate | 12.00 | Met+Cys | 0.75 |
| Soybean oil | 1.90 | Thr | 0.79 |
| Sucrose | 3.50 | Trp | 0.23 |
| Limestone | 0.91 |  |  |
| Dicalcium phosphate | 0.74 |  |  |
| Nacl | 0.25 |  |  |
| *L-*Lys-HCl (78%) | 0.38 |  |  |
| *DL*-Met (99%) | 0.17 |  |  |
| Trp (98%) | 0.05 |  |  |
| Thr (98.5%) | 0.02 |  |  |
| Chloride choline | 0.10 |  |  |
| Vitamin premix^1^ | 0.05 |  |  |
| Mineral premix^2^ | 0.20 |  |  |
| Total | 100.00 |  |  |

^1^The premix provides following per kg diet：VA 5512 IU, VD_3_2250 IU, VE 24 mg, VK_3_ 3 mg, VB_2_ 6 mg, VB_6_ 3 mg, VB_12_ 24 μg, folic acid 1.2 mg, nicotinic acid 14 mg, biotin 150 μg,*D*-pantothenic acid 15 mg.

^2^The premix provides following per kg diet：Fe 100 mg, Cu 6 mg, Mn 4 mg, Zn 100 mg, I 0.14 mg, Se 0.3 mg.

| Table S2 Primes and probes for real time PCR of bacteria | | |
| --- | --- | --- |
| Items | Primer/probe name and sequence(5'-3') | Product length/bp |
| *Escherichia coli* | DC-F,CATGCCGCGTGTATGAAGAA |  |
|  | DC-R,CGGGTAACGTCAATGAGCAAA | 96 |
|  | DC-P,(FMA)AGGTATTAACTTTACTCCCTTCCTC(BHQ-1) |  |
| *Lactobacillus* | RS-F,GAGGCAGCAGTAGGGAATCTTC |  |
|  | RS-R,CAACAGTTACTCTGACACCCGTTCTTC | 126 |
|  | RS-P,(FMA)AAGAAGGGTTTCGGCTCGTAAAACTCTGTT(BHQ-1) | |
| *Bifidobacterium* | SQ-F,CGCGTCCGGTGTGAAAG |  |
|  | SQ-R,CTTCCCGATATCTACACATTCCA | 121 |
|  | SQ-P, (FMA) ATTCCACCGTTACACCGGGAA(BHQ-1) |  |
| *Bacillus* | YB-F,GCAACGAGCGCAACCCTTGA |  |
|  | YB-R,TCATCCCCACCTTCCTCCGGT | 92 |
|  | YB-P, (FMA)CGGTTTGTCACCGGCAGTCACCT(BHQ-1) |  |
| Total bacteria | Eub338F,ACTCCTACGGGAGGCAGCAG |  |
|  | Eub518R,ATTACCGCGGCTGCTGG | 200 |

Table S3 Primer sequences and annealing temperature of pigs

| Target gene | Forward primer 5’-3’ | Reverse primer 5’-3’ | Product length | Annealing temperature (℃) | Accession number |
| --- | --- | --- | --- | --- | --- |
| EGF | ATCTCAGGAATGGGAGTCAACC | TCACTGGAGGATGGAATACAGC | 165 | 60 | NM_214020.1 |
| GLP-2 | ACTCACAGGGCACGTTTACCA | AGGTCCCTTCAGCATGTCTCT | 149 | 60 | NM_005671883.1 |
| GLP-2R | GACCCTCTCTTGTGTCTTCGTA | AAGATGACGTCCTTCGCCAG | 120 | 56 | NM_001246266.1 |
| IGF-1 | CTGAGGAGGCTGGAGATGTACT | CCTGAACTCCCTCTACTTGTGTTC | 137 | 60 | NM_001097417.1 |
| IGF-1R | TTCGCCAGATCCTAGGGGAG | TCCCAGCTTTGATGGTCAGG | 120 | 60 | NM_214172.1 |
| CLAUDIN-1 | ATTTCAGGTCTGGCTATCTTAGTTGC | AGGGCCTTGGTGTTGGGTAA | 214 | 60 | NM_001244539.1 |
| OCCLUDIN | CAGGTGCACCCTCCAGATTG | GGACTTTCAAGAGGCCTGGAT | 110 | 60 | [NM_001163647.2](http://www.ncbi.nlm.nih.gov/entrez/viewer.fcgi?db=nucleotide&id=402746997) |
| ZO-1 | CTGAGGGAATTGGGCAGGAA | TCACCAAAGGACTCAGCAGG | 105 | 60 | [XM_013993251.1](http://www.ncbi.nlm.nih.gov/entrez/viewer.fcgi?db=nucleotide&id=927096856) |
| MUC1 | GTGCCGCTGCCCACAACCTG | AGCCGGGTACCCCAGACCCA | 141 | 60 | [XM_001926883.5](http://www.ncbi.nlm.nih.gov/entrez/viewer.fcgi?db=nucleotide&id=927121447) |
| MUC2 | GGTCATGCTGGAGCTGGACAGT | TGCCTCCTCGGGGTCGTCAC | 181 | 60 | [XM_013989745.1](http://www.ncbi.nlm.nih.gov/entrez/viewer.fcgi?db=nucleotide&id=927100665) |
| BCL-2 | TGCCTTTGTGGAGCTGTATG | GCCCGTGGACTTCACTTATG | 144 | 60 | XM_003121700.4 |
| BAX | AAGCGCATTGGAGATGAACT | TGCCGTCAGCAAACATTTC | 121 | 60 | [XM_013998624.1](http://www.ncbi.nlm.nih.gov/entrez/viewer.fcgi?db=nucleotide&id=927141803) |
| CASPASE-3 | GGAGAACAATAAAACCTCCGTGG | CATCCAAGGATATTCCAGAGTCCA | 101 | 60 | NM_214131.1 |
| CYCLIN D1 | AGCAGGAGCTAAAGCCGAAC | TCGTTGAGGAGGTTGGCATC | 149 | 60 | [XM_013994006.1](http://www.ncbi.nlm.nih.gov/entrez/viewer.fcgi?db=nucleotide&id=927100828) |
| p21/Cit1 | AAGCACAACCCTCAACCACT | TGCAGGTCTGAGAATGCAGG | 112 | 60 | XM_001929558.2 |
| IL-8 | AGTGGACCCCACTGTGAAAA | TACAACCTTCTTCTGCACCCA | 102 | 60 | X61151.1 |
| TNF-α | CGTGAAGCTGAAAGACAACCAG | GATGGTGTGAGTGAGGAAAACG | 121 | 60 | NM_214022.1 |
| IL-10 | GACGTAATGCCGAAGGCAGA | TGCTCTTGTTTTCACAGGGC | 133 | 60 | [NM_214041.1](http://www.ncbi.nlm.nih.gov/entrez/viewer.fcgi?db=nucleotide&id=47524185) |
| GPR43 | TCATGGGTTTCGGCTTCTACAG | GTACTGAACGATGAACACGACG | 197 | 58 | EU122439.1 |
| GPR41 | ACTACTTCTCATCCTCGGGGTT | CTCCACTTCGCTCTTCTTCAGT | 119 | 56 | JX566879.1 |
| β-actin | TCTGGCACCACACCTTCT | TGATCTGGGTCATCTTCTCAC | 114 | 60 | DQ178122 |
